# Book Reviews

**Published:** 1812-09

**Authors:** 


### CRITICAL ANALYSIS
### *OF RECENT PUBLICATIONS*
IN THE
### DIFFERENT BRANCHES OF PHYSIC, SURGERY, AND MEDICAL PHILOSOPHY.

---

*Cases of Apoplexy and Lethargy; with Observations upon the Comatose Diseases. By* J. CHEYNE, M.D. 8vo. Lond. 1812. pp. 224. Five Plates.

DR. CHEYNE is well known to the faculty as an acute and industrious clinical observer; and, by his habit of noting the symptoms of diseases at the bed-side, with careful dissections, our knowledge of the morbid state, in many instances, has been enlarged, and our practice improved.

While employed in collecting materials for another work, an essay on *Hydrocephalus acutus*, the idea of the present was suggested: and, having thenceforward noted every case that had a reference to the pathology of the brain, the practice has terminated in a selection that will tend to make us better acquainted with the nature and treatment of a disease the most alarming and fatal. The author arranges his facts and observations in the following order.

The essay commences with a history of apoplexy; proceeds to the anatomy of the disease, and observations on the morbid appearances. The second section treats of serous apoplexy: the third is occupied in detailing the means employed in the paroxysm: the fourth contains the cases and dissections of apoplexy and lethargy; to which is annexed a commentary on the cases. The volume concludes with a very proper appendage—an explanation of the five plates which elucidate the facts and principles of the preceding pages.

In endeavoring to present our readers with a view of the practical, and therefore valuable, part of this work, we shall not, however, adopt precisely this method, but shall first explain the causes of the two species of this disease, as laid down by Dr. Cheyne; give his description of the appearances that precede the attack, the symptoms in the paroxysm; and conclude with the method of treatment.

The real nature of apoplexy, the causes of the symptoms, and their usually fatal termination, with the most probable mode of preventing or removing the disease, will be best ascertained by the examination of bodies *post mortem*. Of this our author has been fully convinced, and has therefore

 taken taken every opportunity of inquiring into the state of the brain, the organ directly affected in this disease; the result of which he gives in what is denominated the ANATOMY OF APOPLEXY. The state and general appearances of the parts contained within the cranium of those who are apoplectic, are thus described:

"In dividing the scalp, there is often a great flow of blood from the occipetal and frontal veins. During the whole of the dissection, the venous blood flows from all parts of the head: first, from the superficial veins; and then, in a stream, from the sinuses; so that the quantity collected amounts to one or two pounds, or even more.

"The flow of venous blood principally arises from the accumulation of this fluid on the right side of the heart. In the first instance it escapes the more easily, as the veins within the head are not furnished with valves; but it continues to flow after the brain has been taken out of the skull. It is from the turgescence of this part of the vascular system, that the skull-cap is forced up as soon as the section of the bone is complete.

"The dura mater is sometimes thickened and bound to the cranium by the strongest adhesions; and I have seen the small branches of the arteries which supply this membrane, numerous and distinct.

"The tunica arachnoides does not always preserve its transparency; it is sometimes much thickened and opake. The pia mater is sometimes remarkably vascular. The veins are turgid with dark blood; and in particular parts of the pia mater, nay, sometimes over its whole extent, there appears high arterial action; the whole surface acquires a bright vermillion tint, and between the minute and florid vessels, there are particles of scarlet extravasation; the membrane at these parts is bloodshot. There is often observed between the tunica arachnoides and pia mater, a serous effusion, which varies in appearance: in some bodies it is colorless; in others turbid, bloody, or even mixed with streaks of coagulable lymph.

"The substance of the brain is often unusually firm, and, when cut into, the numerous points of blood show that the divided vessels are enlarged. It ought, however, to be mentioned, that, according to Portral, the substance of the brain is more condensed and heavy in aged persons than in early life. 'Chez les vieillards le cerveau est proportionellement beaucoup plus dense et plus pesant que chez les enfans.'

"The ventricles are often enlarged, and contain serous fluid in considerable quantity; and the communication between the ventricles is generally enlarged.

"For the most part extravasated blood is found within the cranium; sometimes between the membranes; sometimes bursting the walls and floor of the ventricles, we find a great effusion of blood both in the substance of the brain and in the ventricles. Coagulated blood has been found in various parts of the cerebrum and cerebellum of the same subject; and it has also been found in the theca vertebrarum. I have never seen extravasated blood on the surface surface of the brain, which did not appear to have risen from a rupture in its substance; but, in a case communicated to me by a friend, the blood seemed to have flowed from a number of the smaller arteries of the pia mater.

"When blood is extravasated into the substance of the brain, it is contained in a cavity, the walls of which are uneven and rugged; and in this irregular cavity the substance of the brain is generally so mixed with blood, that we cannot wash the blood away without carrying along with it portions of medullary matter.

"We do not always discover extravasation of blood, but we never fail to find remains of greatly increased action, and great congestion, in the arterial and venous systems of the brain.

"The membrane lining the ventricles generally exhibits appearances resembling those which are observed upon the pia mater of the surface of the brain. The plexus choroides is not uniform in its circumstances; it is often bloodless, when the ventricles contain serous fluid; but it is also often florid; and not unfrequently there are a number of vesicles attached to it which contain fluid.

"My dissections tend to confirm the observation, that blood is generally found extravasated in the hemisphere opposite to the side of the body paralysed; and that, when paraplegia attends apoplexy, we may expect to find extravasation of blood about the base of the brain, or in the tube of the spine.

"It has been thought, that extravasation of blood is only to be found in the cerebrum. This opinion, however, is erroneous; I have observed extravasation in the cerebellum in three, if not in four, instances.

"Upon dissection, cavities of a large size have been found in the substance of the brain of those who have survived an apoplectic stroke for some time, containing flakes of coagulated lymph, or of bloody-colored fluid, or of limped serum. These cavities have long been attributed to a rupture of the vessels in the original attack of apoplexy."

In the brain of those who die of apoplexy, the most unvarying appearance is, an excited state of the minute arteries of that organ and its membranes; next, an extravasation of blood, the consequence, probably, of the excited state of the vessels; the turgescence of the venous system; the enlargement of the ventricles, partial or general; and, lastly, the serous effusion, which is generally found in various parts of the brain. There are no morbid alterations in other parts of the system appearing to have any connection with the apoplectic state,* the liver excepted, in which

* It is right to state that Morgagni believed the pericardium, after this disease, always contained an unusual quantity of fluid. If this be accurate, says Dr. C., it forms an exception to the general observation of anatomists, that the more sudden the death, the less the quantity of fluid in the pericardium.

Dr.

Dr. Cheyne often found that disorganization, which is the well known effect of the abuse of wine and spirits. It does not clearly appear, however, that, in those instances where this character of disorganisation did occur, there had not been the abuse of wine and spirits; which might have been the exciting cause of the disease of the liver and of apoplexy. If observation shews that this state of the liver, resembling that produced by the abuse of wine and spirits, exists in cases of apoplexy, when that intemperance has not been practised, we will admit a diseased liver of this species, and the altered state of the brain, to have some reciprocal influence on each other. But, as long as intemperance in wine and spirits is as much an exciting cause of apoplexy in certain habits as of diseased liver, we may suppose them both to arise from that source, rather than admit the state of the liver to be indicatory of, or occasioned by, the peculiar disease of the brain.*

The

* In another part of his Essay, p. 175, Dr. C. goes more particularly into the state of the liver, as connected with the apoplectic state; and, as it contains not only ingenious observation, but facts of considerable interest, we shall cite the passage in this note.

"In dissections after apoplexy, we shall seldom fail to find the liver much diseased; and the disease of the liver to be one which, apparently, had been of slow formation. A gentleman of very distinct observation informs me, that he has seen apoplexy fatal in 24 and 36 hours, to patients under ascites connected with diseased liver, in whom the absorbents, in consequence of mercury, had begun to act with great vigor. An interesting case of this nature is related in the 5th number of the Dublin Medical and Physical Essays, by Dr. Mills. The patient, whose liver was hard and enlarged, had been afflicted with ascites and anasarca, but was relieved by the free exhibition of crystals of tartar; but, whilst full of the hope of perfect recovery, he was seized with symptoms of sanguine apoplexy, and was unexpectedly carried off."

This certainly tends to show the influence of the liver upon the sensorium commune. But the observations of our author, on the influence of the hepatic system, do not stop here: in tetanus, in catalepsy, chorea, and epilepsy, and in all diseases of the brain and nervous system, he considers it to be an essential point to look to the condition of the liver. The following statement is too full of interest to be omitted.

"Perhaps, adds Dr. C., I am throwing a ray of light on the subject, when I state, on the authority of an accurate anatomist, Mr. Todd, that, in every dissection he has made after idiotism and mental derangement, and he has made upwards of four hundred, he has found the liver more or less diseased. He observes after insanity generally no great change of color, but the organ is more bulky, with

The *Anatomy of Apoplexy*, as delineated by Dr. Cheyne, is fully supported by his cases. In the 4th case, a coagulum of blood, weighing one ounce and three drams, was found in the left lateral ventricle; and when it was removed, the plexus at the bottom of the ventricle was torn. In the 7th case, when the scalp was divided, there was a great flow of dark blood; and, during dissection, blood flowed from the divided veins to the quantity of two pounds. On the pia mater there were striking marks of inflammation; it was marbled all over with deep red and purple patches of inflamed vessels, and streaks of extravasated blood. Though the substance of the brain had no marks of inflammation in this case, and there was considerable serous effusion, there were many appearances of that state in various parts. The plexus choroides, and velum interpositum, were turgid, and red blood-vessels were in great number on those parts. On the base of the brain, and about the pons varolii, and on the celebellum, the appearance of inflamed vessels prevailed. The 8th case presented a general turgescence and accumulation of blood in the head; on opening the right lateral

---

with a thicker edge, and always connected by preternatural adhesions, sometimes of great extent, to the peritoneum.

"That the brain should be suddenly affected in consequence of its connection with the liver, is not more remarkable than that the liver should be suddenly disordered from affections of the brain. Yet this test is an established observation. I am informed, by a gentleman who has occasion to dissect a great many bodies, that, in diseases of the brain, he never fails to find the liver diseased, either as a cause or a consequence. The same gentleman assures me, that the liver generally discovers marks of recent inflammation after fatal injuries of the head. Every surgeon knows abscess of the liver is a common effect of injuries of the brain.

"Une femme, agée de 80 ans, qui n'avoit jamais eu maladies, eprouva une grande contrarieté, qui lui occasionna un acces de colere si violent, qu'en moins de quelques heures elle fut, prise d'un ictere général d'un jaune trés-force. Le vingtieme jour, il lui survint deux depôts à l'avant-bras gauche. Le vingt-cinquième elle eu des vomissemens bilieux; et elle mourut en repetant, qu'elle ne pardonnait pas l'offense qu'on lui avoit fait.—*Obs. par M. L. V. T. Amand. Bulletin des Sciences Medicales. Avril* 1810.—I cite this as probably the most recent instance on record of an affection noticed by Riverius, the *aurigo ab ira;* and, if I mistake not, jaundice is related to have appeared as the effect of other passions."

At a time when the distant sympathies of the animal system, and the reciprocal influences of organs remote from each other, are looked to with great solicitude, the hypothetical embarrassments connected with this solicitude may be much relieved by these facts.

ventricle ventricle a great quantity flowed out, and in its fore part a coagulum the size of an egg, or larger, was found. The 9th, 10th, and 11th, cases, presented similar appearances.

It appears very decidedly from the facts stated by Dr. Cheyne, that an accumulation of fluid in the brain or its ventricles, either blood or serum, is the immediate cause of the apoplectic paroxysm; and that in all instances, even where the accumulation has been serous, there were accompanying marks of turgescence in the blood-vessels, and appearances, greater or smaller, of inflammation.

The predisponant or occasional causes of this disease, our author states in the following order. 1. Drunkenness. 2. The form of the body. 3. Temperament: sanguine; sanguineo-choleric; choleric. 4. Gluttony. 5. Indolence. 6. Mental anxiety. 7. Fits of passion. 8. External heat. 9. Tobacco. Upon these he makes the following remarks:

"Apoplexy, as appears by this enumeration of its chief causes, is generally the effect of intemperance and improper indulgences. Intoxication, which is entitled to stand the first in the list, may be considered in two points of view in relation to the disease: the habit forms the diathesis; the act is the occasional cause of apoplexy in a great variety of instances: *nay, I believe, the daily use of wine and spirits, even in what is considered a moderate quantity, will lead a man of a certain age and constitution to apoplexy, as certainly as habitual intoxication, which rather leads to mania*, and, when excessive, to a state between phrensy and insanity. My own experience of apoplexy would have inclined me to a different arrangement. I should have placed gluttony—'grandes patinæ et tuceta crassa,' next to drunkenness, and the use of tobacco higher in the scale. It has been observed, if I mistake not, by Cullen, that snuff-taking produces premature senility. The observation, I think, I have seen verified; and I am convinced that apoplexy is one of the evils in the train of that disgusting practice. Form and constitution, when unconnected with intemperance, I should have placed at the end of the list. It is well known that deep study, and despondence, in consequence of great misfortunes, have occasioned apoplexy."

Having stated the remote and proximate causes of apoplexy, we shall proceed to a short detail of the phenomena produced by those causes: 1. As indicatory of the approach of the apoplectic paroxysm; and, 2. As descriptive of the paroxysm itself.

The appearances of the apoplectic paroxysm are so marked that few will misunderstand them; however graphic, therefore, the description of Dr. Cheyne, and faithful to nature it is in the highest degree, we think it of more importance to dwell on the symptoms that precede or mark the approach of the disease. These we consider to be of importance, because, because, by a due attention to them, the impending danger may often be obviated, and the patient rescued from the attack of a fatal malady.

"Some of the appearances observed before the apoplectic fit, which are vulgarly thought to promise long life, are, in reality, the effects of disease. The fulness of the body is often attended with debility of the bronchial membrane, as evinced by wheezing, and frequent attacks of catarrh. There is a languor, which stimulating liquors confirm, although they relieve it while their immediate effects last. There is an inactivity and muscular debility, which few men advanced in life are willing to acknowledge. And, to the discerning observer, the ruddiness of the complexion often demonstrates a diseased state of the cutaneous veins, connected with morbid actions of the stomach and liver. *Bona sua debent suspecta habere*, is the caution of Celsus; and it ought to be repeated to every person past forty, of a full habit of body, who lives intemperately.

"Many of those fallacious perceptions which are known to arise from increased activity of the circulation in the head, have been observed for some time before a stroke of apoplexy; such as muscæ volitantes, tinnitus aurium, and various modifications of vertigo; and along with these there is often epistaxis, and a feeling of a weight or tightness, or a tensive pain across the forehead, or painful throbbing felt on either side of the tuberosity of the occipital bone, and flushing of the countenance, with heaviness or wateriness of the eyes. These symptoms are often in excess. Temporary fits of blindness, and unusual flashes of light, of a bright or shining redness, are complained of. Some have had the sensation of loud and discordant noises, like the boiling of an immense cauldron, or like the roaring of the sea, or the clamours of an unruly crowd. The nights are often restless, from anxiety, palpitations of the heart, and frequent returns of incubus. On the contrary, the patient is sometimes lethargic, sleeping longer than usual, and dozing during the day. His articulation is less distinct, he has no mental energy, he is forgetful, timid, irresolute, and confused. Various slight paralytic affections occur, as weakness of parts of the body, sometimes on one side; spasms of particular muscles, and numbness in the course of particular nerves. Before the apoplectic fit we may often discern great disorder in the chylopoietic viscera; and, indeed, all the symptoms which indicate a constitution impaired in every vital organ. But it cannot be denied, that apoplexy sometimes seizes those whose health, to every appearance, was unbroken, and who had felt unusually vigorous for some time before the attack.

"The attack of apoplexy is often instantaneous, but it is also gradual. Sometimes confusion of ideas, flushing of the face, headachs, nausea, drowsiness, and starting in sleep, are observed in succession; and these symptoms, though they indicate the greatest danger, as prognostics of a confirmed apoplexy, have often subsided from regimen alone, and are generally to be relieved by immediate assistance."

 Beside

Beside these predisponant and proximate causes of apoplexy, physicians of great reputation have supposed some atmospherical influence to have produced an epidemic constitution, productive of apoplexy. Thus Baglivi, Lancici, and Morgagni, explained the frequent occurrence of apoplexy in some years. In May 1721, and in 1741, the frequency of this disease was explained from the sudden recurrence of heat after cold. The great heat of 1808 seemed to be productive of apoplexy. Our countryman, Dr. Cole, whose treatise was published in 1689, endeavors to make the great increase of apoplexy in 1684 depend on the debility produced by the intense cold of 1683.

The predisponent and proximate causes acting to a certain degree, the apoplectic paroxysm is produced. But for a description of this we must refer to Dr. Cheyne's book.

The treatment of the disease during the paroxysm is confined to, 1. Blood-letting. 2. Emetics and purges; and, 3. External applications. All these Dr. C. examines with critical minuteness, more especially the first and second. Of blood-letting, he is the decided advocate. His opinion is founded on the state of brain in those who die apoplectic, and is ably supported. To do it justice, the whole section should be quoted. In deciding upon the propriety of blood-letting in the paroxysm, as the embarrassment of the practitioner arises principally from the notion that by it the serous species might be aggravated, or the probability of subsequent paralysis increased, it may not be useless or improper to give a summary of the experience of our author upon this important point.

"I believe I speak within bounds," he observes, "when I say, that I have attended fifty fatal cases of apoplexy, in which there was room for practice. Of these only one was a case of serous apoplexy, the rest, to every appearance, were sanguineous: the nature of many of these cases was demonstrated by subsequent dissection. Now, admitting this proportion, that there are fifty cases of sanguineous apoplexy, which require the physician's assistance, for one of serous, (and as some distinguished physicians have altogether denied the existence of serous apoplexy, it is probable that I have not overrated the proportion,) we may found an argument in favor of bleeding, which will not be easily overturned, upon the relative frequency of the two species of the disease."

It appears, on the evidence produced by Dr. Cheyne, that what may truly be called serous apoplexy, is a disease of very unfrequent occurrence. He questions if the cases produced by Morgagni were more than three of the twenty that belong to serous apoplexy; and even against them, as perfect specimens of that species of the disease, much objection lies.

lies. The great object, however, though serous apoplexy may be a rare disease, as it is admitted sometimes to have happened, is to distinguish between it and the sanguineous species. But here we are left in a state of incertitude. It would be inferred that pale, emaciated, and relaxed, persons, in advanced life, who have lived abstemiously or temperately, would be the subjects of serous apoplexy; but, when such persons have died apoplectic, the vessels of the brain have been found gorged with blood, or there has been extravasation of that fluid. The learned pathologists Morgagni and Portal do not give any satisfactory information. The conclusion then is, that, as we want diagnostic marks by which to distinguish the species, and as the cases in which accumulation of blood takes place in the brain are as fifty to one, (on the evidence of dissection,) we should abstract blood in all instances of the apoplectic paroxysm, as the safest alternative. To this Portal assents, when he recommends the same treatment in both species. This is, however, a point of too much importance to be hastily decided on; we should be glad to see the question still further argued; and also a more determined prophylactic system of treatment laid down.

The use of emetics and purges, especially the former, have excited professional controversies, which have explained little more than the unsettled opinions upon the nature and treatment of apoplexy, and the unphilosophical spirit which actuated the contending parties. Dr. Cheyne is decidedly against the use of emetics, and gives his reasons at large. He examines and controverts the arguments of *Pyrrho*, as they appeared in this Journal in the controversy between Crowfoot and Langslow.

Upon external applications not much is said. Blistering the scalp, at least at the commencement of the paroxysm, is reprobated; but the application of cold to the head is recommended, by sponging with water and vinegar, solution of muriate of ammonia, or iced water.

The connection between apoplexy and other diseases of the brain and nervous system, as catalepsis, extasis, caros, cataphora, lethargy, &c. appeared to Cullen to be so close, that he reduced them to one genus, Apoplexia: and accordingly, in his Nosology, they appear but as species of that affection. The truth and propriety of this Dr. Cheyne denies; and argues very forcibly both on his own experience and observation, as well as from the statements and opinions of many other physicians, for the necessity, if we attend to the phenomena of these diseases as they appear in nature, of making them distinct genera. The whole of these observations are well deserving attention, but we have already given so much space to this article, that we must refer our readers to the work itself, with an assurance that they will be recompenced for their trouble. We cannot refuse, however, to quote the following passage, as containing some facts in confirmation of a principle supported with much ingenuity by a physician of Montpellier.

"The annals of medicine abound with instances of metastasis and comersion in the diseases of the skin. Thus the exanthematous eruptions, when suddenly repelled, are followed by hurried and oppressed respiration: tinea has ended in hydrocephalus, and erysipelas in lethargy. Several cases of this kind are annexed to Hoffman's Thesis de Affectibus Soporosis. One of them is the case of a corpulent and plethoric man, of fifty years of age, of a sanguine temperament and bad habit of body, who had an erysipelas with shivering and fever. On the third day of this disease he allowed an empiric to apply to the affected part an epithora of comfrey boiled in vinegar; immediately after which he fell into a deep sleep, from which there was no rousing him. He was let blood, glysters were administered, and nitro-camphorated powders given, and soon after the patient was roused from his sleep; but he, who had been of the most lively parts, and had possessed an incomparable memory, had his mind entirely destroyed. His memory had suffered so much, that he could not recollect any thing which had happened to him. Every remedy was tried in vain. He remained several years in this deplorable state; during which his appetite continued unimpaired, and his body was duly nourished.

"When a patient, after a pleuritic attack, dies of carus, he is delirious before he sinks into stupor. This is also the course after ischuria. In some rare instances of coma after ischuria, it would appear as if the vessels of the brain assumed an action similar to the healthy action of the vessels of the kidney: fluid, with the color and smell of urine, having been observed in the ventricles of the brain. J'ai, reconnu l'odeur d'urine dans une épanchment d'eau qui s'étoit fait dans les ventricles du cerveau d'une homme mort d'une suppression d'urine. *Portal.*—And yet this is scarcely more extraordinary than the case of the girl whom I saw in 1787. She vomited, at stated times, a fluid with all the sensible qualities of urine; the discharge by the urinary passage having long been suspended."

Upon the extraordinary conversions, either in structure or functions of various parts of animal bodies, Dumas has a dissertation of considerable research, in which will be found many facts similar to the above.

We must here take leave of this valuable addition to practical medicine, written upon a class of diseases of the first importance, and in which the most prompt treatment is required. We confess to have risen from its perusal with much much clearer notions of the nature and management of Apoplexy, and with this conviction we recommend it without reserve to the profession.

---

### *Edinburgh Medical and Surgical Journal, No.* XXX.

(Continued from p. 150 )

X. *Some Remarks on the Fevers of Sicily; with an Account of the Autumnal or Bilious Remittent Fever of that Island, as it appeared among the British Troops; by* ALEXANDER BOYLE, A.M. Surgeon.—This is an elaborate, perhaps verbose, paper on the Fevers of Sicily, by a gentleman who, having resided five years on the island, seems, so far, well qualified to write on its diseases. An essential step toward understanding the diseases of a country, that is, becoming acquainted with the vicissitudes of its seasons, and the apparent effects the changes produce on the animal fabric, was judiciously taken by Mr. Boyle. On this subject he says,

"The vicissitudes of seasons, and the various modifications of atmospheric constitution, powerfully influence the character of diseases, and the appearance of certain epidemics, in this climate. Accurate meteorological observations, therefore, as well as the investigation of those circumstances, which properly constitute medical topography, present an interesting field of inquiry, and not only facilitate the investigation of the cause of diseases, but form a branch of knowledge essentially connected with their prevention, and method of cure.

"In remoter ages, this subject received particular attention, and almost every page of the writings of Hippocrates inculcates the utility of this study,—which was considered, by that great man, not less essential to the physician, in order to enable him to predict approaching changes, and guard against their effects, than it was deemed necessary by the Mantuan poet, for the husbandman and mariner.

"Hinc tempestates dubio prædiscere cælo
Possumus; hinc messisque diem tempusque arendi;
Et quando infidem remis impellere marmor,
Conveniat, quando armatas deducere classes
Aut tempestivam silvis evertere pinum.
Nec frustra signorum obitus speculamur, et ortus,
Temporibusque parem diversis quatuor annum."

"As far as regards the great revolutions of the year, and the periodical return of the seasons, a great uniformity obtains; and the observations of one year on this head, with but little variation, apply almost to every other.

"On this depends the great uniformity of the diseases which usher in the different seasons. Those seasons, however, differ materially from each other; and a difference not less remarkable, and important in practice, characterises the diseases by which they are invariably bly attended, and which, agreeably to those changes, undergo certain alterations of type, as well as of other important symptoms.

"The occurrence of the dry and rainy seasons may always, indeed, be expected with tolerable certainty, at the regular and accustomed periods. The diurnal vicissitudes, however, are very frequent, and, until the hot season has fairly set in, sudden changes of temperature are very common.

"From the beginning of June, may be dated the commencement of the hot season; from which time, until the beginning of September, the temperature continues pretty steadily about the same standard, and, as observed in the shade at noon, usually ranges between 82° and 86° of Fahrenheit's scale. During this period, east or westerly winds for the most part prevail; rain seldom falls; and it frequently happens that the months of June, July, and August, pass away, even without a single shower. Of these, July is the hottest month, and the thermometer sometimes reaches the 89th degree; while the suffocating sirocco winds commonly continue until noon; after which hour a westerly breeze generally springs up.

"About the latter end of May, inflammatory affections of the head begin to make their appearance; but they, as yet, are generally slight. In the month of June, however, and as the heat of the season advances, they become more frequent in their appearance, as well as more violent in their symptoms.

"About the beginning of September, the weather undergoes a very remarkable change, which is more particularly to be attended to, on account of the important influence it exerts as a cause of the disease which now succeeds. Together with a diminution of temperature, rains begin to fall, accompanied with thunder and high winds. About this time, the thermometer falls to 76°, indicating a diminution of temperature, not less than 10 or 12 degrees. This change is sudden, and produces a very conspicuous effect on the nature of the prevailing disease.

"The fevers still are of the inflammatory kind, but the affection of the head no longer forms so conspicuous a symptom; instead of which, they now manifest themselves under the form of phlegmasiæ affecting other parts.

"The showers however are but transient; the mercury in the thermometer again rises, but does not again attain so great a height; and the weather becomes dry and settled, until about the end of October, when heavy rains set in, accompanied with a considerable and permanent diminution of temperature, which puts a period to the violence and frequent occurrence of the epidemic.

"During the winter and spring, pneumonia and acute rheumatism are the prevailing complaints; but those which prove most tedious and fatal are obstinate dysenteries and intermittents; the common sequel of violent attacks of the autumnal fever, and dependent on chronic affections of the abdominal viscera.

"On the approach of autumn, the character of the fever, as has been already said, undergoes a considerable change; and, though this this is effected by apparently slow gradations, one epidemic passing, as it were, imperceptibly into another, they not only differ essentially as to their exciting causes, but also in their origin, and seat; the febrile disease of summer arising from affections of the encephalon, depending on inflammation and its consequences; while those of autumn are more particularly dependent on inflammatory affections of the stomach, and other abdominal viscera."

The fever which makes the subject of this paper, being connected with, or depending on, an inflamed state of the encephalon, or of the stomach or other abdominal viscera, according to the season of attack, blood-letting, and other evacuants, were alone to be depended on. With a view to obviate or to remove debility, as the disease assumed an intermittent form, *cinchona* was sometimes employed: "but I feel myself warranted (says Mr. Boyle) to affirm, from the result of several cases in which this plan was adopted, in the fever now under consideration, that *bark* served only to exasperate the local disease, and to aggravate every symptom of the succeeding paroxysm."

Mercury, especially in the form of the sub-muriate, the author found to be so effectual, that, he says, it is to be resorted to immediately, both externally and internally, as the most powerful remedy we possess in the treatment of this disease.

XI. *Case of singular Affection of the right Leg, which, on Extension of the Limb, excited agonizing Head-ache; by* James Ramsey, M.D.—A young lady, eleven years old, with feverish symptoms, debility, and loss of appetite, constipated bowels, foul tongue, and offensive breath, had a singularly acute pain in the head, and flexor muscles of the right leg, whenever that limb was in the least extended. It was presumed that the cause of all the complaints was in the head, and indicating an approach to hydrocephalus. Leeches were applied to the temples, active purges were persisted in, with blistering, and digitalis. Under this management the disease soon gave way, and the patient was restored to perfect health.

XII. *Case of Poisoning, by the external Application of Corrosive Sublimate; by* Archibald Robertson, Surgeon.—A solution of four or five grains of the muriate of mercury to an ounce of alcohol, was freely applied, morning and evening, to the eruptions of psora for five days. On the seventh day the patient, the man, became extremely feverish, with intense head-ache, flushed countenance, full and quick pulse, and great heat on the skin. In the night, violent pain in the stomach, with vomiting and painful retching, came on, continuing the whole night. In the morning succeeded ceeded diarrhœa and tenesmus, with the evacuation of a small quantity of bloody mucus, and extreme debility, which was followed by severe ptyalism. Sudorifics, opium, and nutritious diet, recovered him. The principal point in the case is the action of the poison on the first passages, though taken into the system by the cutaneous absorbents. A short time since, we related a similar instance of affection of the first passages, from the application of a nostrum to the arms, in which was a strong solution of *hydrarg. murias.*

XIII. *Case of Palsy cured by Titillation; with some Observations on the Effects of Titillation on the Nervous System; by* James Wardrop.—From the novelty of the remedy, the fortunate result, and the ingenuity of Mr. Wardrop's remarks, we are induced to give our readers the principal part of his detail.

"A man twenty-three years of age, in the month of April, 1810, when with the army in Portugal, was seized with a fever, which was at that time epidemic among the troops. In consequence of repeated attacks of the disease, he remained for nearly twelve months in the different military hospitals of Coimbra, Lisbon, &c. Besides what he described as the febrile disease, he had a severe affection of his head, for which he was bled profusely; and, when he returned to Britain, he had a complete paralytic affection of the left side. By living in the country, he regained a good deal of strength; and he was able to move, though in a very small degree, the paralytic arm and hand. Soon after this, the complaint in his head returned with its former violence, but, by copious and repeated bleedings, the giddiness and stupor, which were the chief symptoms, were removed. The paralytic affection of the left side, during this attack, became more complete; and, although his general health improved, yet, in the beginning of August last, notwithstanding the most rigid antiphlogistic treatment, and a continued use of blisters and electricity to the affected side, the limb continued feeble, and his arm and hand perfectly useless.

"When I saw him at this period, which was the middle of August last, eighteen months from the commencement of his illness, he had a marked halt in the left leg, and the left arm hung motionless, not having the power of even producing any sensible movement with any of his fingers. The arm, too, was considerably wasted; the hand œdematous, with occasional pains about his shoulder; his pulse a little full and frequent; his tongue furred, and a tendency to headache. After completely emptying his bowels by aloetic purgatives, and cupping him in the neck, by which treatment all the febrile symptoms, and those of determination of blood to the head, seemed to be removed, I began to try the effect of *titillation.*

"The process which was employed to titillate was extremely simple. A person was directed to draw a feather gently over the surface of the skin on the palm of the hand, until it excited laughter; and I purposed that this should be done three or four times a-day.

"On

"On trial, it was found that the desired effect could readily be produced. At first it was found that a considerable time was necessary to excite laughter; but, the more frequently the application was employed, the more quickly it was accomplished. He found, too, that the laughter was produced much more speedily when he was tickled by another person, than when he used the feather himself; and that it was most easily excited, by applying the feather to the part of the palm where it is marked by the flexion of the thumb.

"The beneficial effects which the titillation produced on the paralytic arm, exceeded my most sanguine expectations.

"After employing it a few days, he began to feel as if the limb were re-animated; a sensation which lasted a short time after each fit of laughter. This sensation was soon followed by a power to produce a sensible and voluntary motion of the fingers, which daily increased; so that in about a month from the commencement of the use of the titillation, he could grasp his hand with a moderate firmness, and move, in like manner, the elbow and shoulder joints. After this period, the strength of the limb was rapidly regained; and in two months from the commencement of this treatment, I met him in the street carrying a bundle under the affected arm.

"The titillation was also used to the lower extremity of the affected side, the feather being applied to the sole of the foot, where it failed not to produce a speedy and great degree of laughter.

"Besides the titillation, it ought to be mentioned, that I also advised him to rub the limbs with the dry hand, morning and evening; and this was done with a view not only of removing the œdematous swelling, but also of producing an additional excitement on the skin. I have seen the patient four months after the above treatment had been employed, when he remained perfectly well. I am aware that the event of any new mode of treatment, in an individual instance, cannot be sufficient to warrant any general conclusion. The case, however, seems to me to afford an interesting illustration of the influence which the functions of the skin have on the nervous system, and to open a channel for observation and experiment, in various diseases of that system, which may lead to important improvements in their treatment. Under that impression, I have ventured to lay these remarks before the public, in their present form; and with the hopes that, by their early communication, the good effects of the practice may be established by the ample experience of others.

"It may be remarked, before concluding these observations, that a good deal of discrimination must necessarily be employed in selecting cases where titillation is likely to prove beneficial, and in determining the extent in which it may be prudent to employ such a remedy. As long as the paralytic affection seems to depend on an increased quantity of blood in the encephalon, or to arise from any morbid alteration in the structure of the brain, or its coverings, all attempts by titillation, or other means, to recover the power of the paralytic nerves, must be fruitless, if not highly dangerous. Titillation appears to me to be applicable to those cases only where the primary affection is removed, and where the palsy, which was merely one of its symptoms, alone remains. How far titillation may have an effect in alleviating the excruciating pain of *tic douloureux*, sciatica, and some other affections which seem chiefly confined to nerves, experience can alone determine. Titillation, when employed in the treatment of diseases, must, like other powerful remedies, be at first used with caution; and we are enabled to vary the extent of its effects, from tickling to laughter, or even convulsion.

"Every one knows, from personal experience, the two first degrees of its effects. There are, I believe, many instances of its having produced convulsions, and I have heard of one case where it proved fatal. In all probability, the effects of titillation, as a remedy, should be confined to the excitement of laughter, which in old or obstinate cases may be excited even to an immoderate degree.

"It is, perhaps, difficult to determine how far titillation acts directly on the affected nerves, or indirectly through the medium of the brain; the latter appears to be the most probable opinion; and, if this be found to be the case, it may be equally beneficial to titillate the skin of the sound as of the paralytic limb, thus obtaining a more extensive, and a more efficacious, mode of applying titillation as a remedy. The influence of mental emotion on the nervous system, and on the vital and animal functions, has not yet met with that consideration in the treatment of diseases which it appears to merit. The effects of music in some of these diseases, and the variations of the same disease in individuals, under the influence of different mental emotions, prove this influence, and might lead us to expect that great beneficial advantages might be derived, from acting on the minds of those afflicted with such complaints.

---

*An experimental Examination of the last Edition of the Pharmacopœia Londinensis; with Remarks on Dr. Powell's Translation and Annotations. By* RICHARD PHILLIPS. 8vo. pp. 148. London. 1811.

We have to apologize for the delay which has occurred in our account of this publication, which unquestionably merited an earlier notice. In one respect our neglect may be advantageous. The last edition of the London Pharmacopœia has incurred much criticism, and in our opinion the objections that have been urged, not having been regarded in the proper quarter, ought to be revived. When the new edition was announced, the great note of preparation, the issue of a specimen *primum et alterum*, previous to the appearance of the *magnum opus*; the improved state of chemical science; the recent publications of the colleges of Edinburgh and of Dublin; all combined to raise anticipation of the London edition to a high pitch. In a work of this nature it were vain to expect any thing like perfection, the art of chemistry is still progressive, and we must admit that the new edition of of the London Pharmacopœia, considering the immense distance the former one was behind the present state of the art, has made a very successful attempt at overtaking it. Still the work is extremely incomplete; and, unfortunately for the reputation of the college, much of the imperfection might have been avoided. The fellows, indeed, did invite the licentiates to contribute their remarks on the occasion, and in some instances adopted the suggestions that were furnished by this degraded class of the profession. *But* we did not hear that any of the licentiates who had devoted themselves more especially to chemical pursuits, were invited to co-operate with the committee appointed by the college for conducting the experiments, and preparing the new Pharmacopœia; neither could we learn that any professors of chemistry, practical chemists, or men of science, with or without degrees, were consulted, although it is well known that the names of our first chemists are not recorded in the college list.

The natural consequence of this proceeding was immediately exposed by Dr. Bostock and other writers; amongst whom none is more conspicuous than Mr. Phillips, who has followed the committee in most of their experiments and preparations. We doubt not that, had this gentleman been properly applied to in the first instance, much of the confusion and error which now render the Pharmacopœia a dangerous guide, would have been obviated. It is hardly credible that, notwithstanding the practical errors committed in the directions for preparing some of the compounds, the absurdities in the nomenclature, the improper retention of some articles, and the undue rejection of others, with the frivolous change in the power of certain medicines, have been forced upon the attention of the college from various quarters, the Pharmacopœia is still permitted to disgrace that learned body, and to produce perplexity and mischief in practice. A new edition was urgently demanded, and the first, however handsomely and expensively printed, ought to have been called in; even now it is not too late; for many practitioners, deceived and disappointed by some of the new processes and preparations, have yet recourse to the old Pharmacopœia.

Having offered these general remarks, not from any ill spirit, but with the sincere desire that the college might yet retain its high station in public opinion, and prevent any further ill consequences from the officinal preparations being made according to its directions; it becomes us to support our case by facts, of which the work before us presents a sufficient quantity: we shall content ourselves with citing only a few. In the very commencement of the work it is curious to observe the college violate its own rules. Thus *octarius* is given us as a new term to express a pint by measure, while the old term *libra* is intended to denote a pound by weight; but, as some fluids are weighed, in the first instance (viz. the 4th process,) that this occurred in the Pharmacopœia, *libra* was preceded by the word *pondere;* yet, in the same process, *selibra* also occurs applied to a fluid, and the operator is left to guess whether that fluid is to be weighed or measured. Many other instances of this sort of inaccuracy are enumerated by Mr. Phillips.

In the preparation of the acids several errors of consequence appear. The alteration of strength in the diluted sulphuric and nitric acids, though trifling in itself, is yet perplexing to the practitioner, who may not be aware of the change, and certainly was needless.

Among the alkaline preparations, after noticing some most unfortunate blunders and inconsistencies, Mr. Phillips observes,

"Liquor ammoniæ is now directed to be used in many of those preparations in which the subcarbonate was formerly employed; and it is particularly requisite that the attention of practitioners should be directed to this circumstance. I have already noticed the great difference which exists between the strength of the former aqua ammoniæ puræ and the present liquor ammoniæ; the power of the linimentum ammoniæ fortius, and of the linimentum camphoræ compositum, has also been greatly increased. It is, however, much more important to point out the extravagant increase of power added to those medicines which are internally employed, viz.

Sp. Ammoniæ.

——— aromaticus.

——— fœtidus.

——— succinatus.

Tinctura Valerianæ ammoniata.

——— Guaiaci ammoniata.

"With the exception of the preparation last mentioned, the quantities assigned by Dr. Powell for the doses of these medicines, are equal to those of the former weaker preparations given by Dr. Latham. I cannot quit this part of the subject without remarking, that, of all the improvements attempted by the college, their failure in this is calculated to produce the most extensive mischief; and I will venture to assert, that scarcely one of the preparations above mentioned could be exhibited in the doses stated by Dr. Powell, without danger to the patient, and disgrace to the practitioner."

As this subject is of the deepest importance, we shall cite some further observations of our author from another part of his book, under the head Spiritus Ammoniæ, et Spiritus Ammoniæ Aromaticus.

"In

"In the Pharmacopœia of 1787, spirit of ammonia was prepared by adding three pints of proof spirit to a mixture of four ounces of muriate of ammonia, and six of impure subcarbonate of potash: one half of the spirit, being distilled, constituted the spirit of ammonia: and the compound, or aromatic spirit was prepared by adding oils of lemons and cloves to a portion of this product. These processes are both objectionable; for, three parts of subcarbonate of potash not being sufficient to decompose two of muriate of ammonia, a part of the latter salt must be wasted; the loss of spirit is however still more considerable. Proof spirit is composed of about 19 parts of rectified spirit and 13 parts of water; three pints consequently contain about 28 fluid ounces of the former, of which 24 only were directed to be distilled. The compound spirit was not only colored, but frequently turbid, owing to the usual impurity of the oils of lemons and cloves. The modes of preparing these medicines have now undergone considerable alteration, and the new processes derive from novelty their only merit, and from mischief their sole importance.

"The following are the directions for preparing the Spiritus Ammoniæ: Take of rectified spirits, two pints; solution of ammoniæ, a pint. Mix.

"The Spiritus Ammoniæ Aromaticus is prepared by adding a fluidrachm of oil of lemons and oil of cloves to a pint of the simple spirit.

"Dr. Powell asserts, that 'alkohol dissolves ammonia, but not its carbonate; water dissolves both; and it is further intended, that the present spirit shall be of sufficient concentration to dissolve certain volatile oils in a subsequent preparation. The former preparation,' he continues, 'appeared very uncertain as to strength, and a large portion of carbonate of ammonia sublimed over which was not dissolved; the object of the present change has therefore been to obtain a more definite article than the former.'

"Now the first of the above-quoted allegations is proved to be erroneous by a very simple experiment. I have stated that, in the former preparations, only 24 of 28 parts of the rectified spirits directed to be used, are obtained by distillation, the product must therefore be considered as very highly rectified spirits; and yet, if some of it be added to a solution of muriate of lime, a copious precipitate of carbonate of lime ensues.

"Neither have I observed that the former preparation was at all uncertain in its strength; but, on the contrary, the results of my experiments coincided to prove a very sufficient degree of uniformity in this respect, as I shall presently more particularly shew; whereas the present process has, in addition to its other faults, that of uncertain power in an eminent degree. Under the head of Liquor Ammonia, I have stated, that I found its sp. gr. to be ·904, whilst that from Apothecaries' Hall had a sp. gr. of ·988, and the power of the first of these preparations is nearly five times as great as that of the latter; consequently, the aromatic spirits of ammonia, prepared with solutions varying as these, will differ equally in power.

3 "In

"In order to compare the strength of the new and the former preparation, I again made the liquor ammoniæ, but its sp. gr. instead of being as before ·904, was ·914, and it was consequently weaker than that which had been previously obtained. With this I made the spiritus ammoniæ aromaticus as now directed, and added a fluid ounce of 'it to a portion' of muriatic acid, which previous experiments had shewn to be capable of decomposing 240 grains of marble; the solution was strongly acid, and I therefore added some pieces of marble to it, having first noted their weight. After heating the solution to ebullition for a considerable time, it appeared, on drying the undecomposed marble, that 145 grains had been dissolved; consequently the saturating power of a fluid ounce of the spiritus ammoniæ aromaticus is equal to that of 95 grains of marble. This experiment was repeated with scarcely any variation.

"I now treated the spiritus ammoniæ compositus of the late Pharmacopœia in a similar way, and the mean of two experiments, which varied but little, shewed that its saturating power was only equal to about 32 of marble, and consequently, that the new preparation, when made with liquor ammoniæ of sp. gr. ·914, which is far from being the strongest which the collegiate process is capable of yielding, contains almost precisely three times as much ammoniæ as an equal quantity of the former medicine. Nor is this all: the ammonia in the new preparation, not being combined with any carbonic acid, is rendered much more acrid and powerful; and the difference between the doses of the two preparations is consequently still greater than that of their saturating power. Notwithstanding this vast difference, Dr. Powell has stated the dose of the new preparation to be one fluidrachm; which is equal to that of the former preparation mentioned by Dr. Latham. But it is still more extraordinary that Dr. Powel should have stated the dose of tinctura guaiàci ammoniata, and of tinctura valerianæ ammoniata, to be two fluidrachms; for, unless it be granted that guaiacum and valerian destroy the power of half the ammonia, this quantity, even upon Dr. Powell's own authority, must contain a double dose; and it follows also, that in giving two fluidrachms of the present volatile tinctures, six times as much alkali must be exhibited as in a fluidrachm of the former spiritus ammoniæ compositus, and that the difference in power would be greater even than this."

Mr. Phillips then shews how the process may be modified so as to obviate his objections, and concludes his observations on this preparation with stating, that an instance has fallen under his notice "in which strangulation was nearly induced by the exhibition of the new preparation; the physician who prescribed it not being aware of its vast accession of power, and of its extreme causticity."

In the preceding account we are satisfied there is no exaggeration. We have ascertained that the spiritus ammoniæ aromaticus prepared by different chemists varies considerably in strength, and, if the college directions are strictly adhered to, to, is so extremely caustic, that one drachm requires at least six ounces of water to dilute, before it can be swallowed. Half a drachm given in a draught of an ounce and a half of mint water nearly proved fatal to an elderly lady. Dr. Powell gives the full dose of tinctura valeriana ammoniata at two drachms; yet one drachm and a half, in a six ounce mixture, was too acrid to be swallowed without exciting extreme uneasiness and pain. Surely he could not be acquainted with the nature of the composition, of which he directed a dose impossible to be taken in a draught of the usual quantity of fluid menstruum.

Of Spiritus Ammoniæ Succinatum, Mr. Phillips remarks,

"In Dr. Powell's table it is stated, that 30 minims of oil of amber are a dose, and in this preparation, in order to give 1 minim of the oil, 2¼ fluid ounces of liquor ammoniæ must be exhibited: with a dose of oil of amber, therefore, 1800 of Dr. Powell's doses of liquor ammoniæ are given; a quantity which, reduced to the strength of the old preparation, as made at Apothecaries' Hall, was considered by the College in 1787 as sufficient for twenty-five doses a-day for a twelvemonth; and Dr. Powell's dose of this preparation contains scarcely 1-1300th part of the quantity he assigns for the dose of oil of amber."

Under the head "Antimonii Oxydum et Antimonium Tartarizatum," Mr. P. has pointed out some egregious and dangerous blunders, which, however, our limits will not admit of detailing.

It might have been supposed that more than usual care would be taken in the preparation of liquor arsenicalis, yet it appears from our author's statement, that

"The mode of preparing this extremely active medicine is very loosely and imperfectly described by the college, and rendered yet worse by the translator. The oxide of arsenic and subcarbonate of potash are to be boiled together in a pint of water until the arsenic is dissolved; four fluidrachms of compound spirit of lavender are then to be added to this solution, and, lastly, a quantity of water, sufficient to occupy with it a pint measure.

"Now I have found (continues Mr. P.) that the arsenic has been completely dissolved before even two fluidrachms of water were evaporated, and consequently, if four fluidrachms of spirit of lavender had been put to the solution, the intended quantity would have been exceeded, without the addition of any water. A certain and rational direction would have been, that the ebullition should be continued until a specified portion of water was evaporated.

"Dr. Powell has rendered the directions very incorrectly in both editions of his translation. In the first he says, 'add as much water as may exactly fill a pint measure.' In the second edition it is altered to—'add as much more distilled water as may be requisite to make up a pint measure;' but, as it is not stated in either version that it it is the arsenical solution and the water to be added to it, which are *together* 'to fill' or 'make up a pint measure;' the words made use of are merely circuitous expressions for—add a pint of water to the solution. It is evident, therefore, that, by observing these injunctions, the solution of arsenic is reduced to about one-half of its intended strength; but those very errors have probably been productive of safety, by preventing the fatal effects of some most gross and unpardonable blunders which Dr. Powell has committed, with respect to the doses of 'arsenic, the most virulent of the mineral poisons.' He states, and, if the solution be prepared according to the directions of the text, states almost correctly, that 'each ounce,' or, as he ought to have said, each fluid ounce 'contains four grains of oxide of arsenic;' and then adds, that 'each drachm,' being one-eighth of an ounce, 'contains one-eighth of a grain;' instead of one-half of a grain of oxide. It is nauseating again to notice this monstrous error which has been so much animadverted upon: it is however requisite, because, in the prodigious list of errata, of which list, I know not whether to say fortunately or unfortunately, copies were with difficulty to be procured, this mistatement, although noticed, is not rectified, but merely diminished; the words 'fluid half drachm' being directed to be substituted for drachm. It is however evident, that a fluid half drachm, instead of containing, according to this new reading, one-eighth of a grain, contains one-fourth."

We think we have now stated enough from Mr. Phillips's able performance, to prove the necessity of the present edition of the London Pharmacopœia being immediately called in, and a more perfect one issued; and, as a translation is considered to be necessary, we hope the future editor will take due notice of Mr. Phillips's concluding lines, with which we close our notice of his valuable work.

"I shall conclude these observations with remarking, that the posological table annexed by Dr. Powell to his translation, is very imperfect; and I have occasionally adverted to instances of incorrectness requiring careful revision. The table also expressing 'the relative value in avoirdupois weight of various weights troy,' is one unvaried mass of error: it seems to have been intended for the use of those persons who prepare medicines in large quantities, and it has probably been productive of many important mistakes. These fundamental errors have been the occasion of this collection of misstatements; first, the avoirdupois drachm is reckoned to contain 27·975 grains, instead of 27·34375; secondly, the avoirdupois ounce is estimated to consist of 8 drachms, instead of 16; and, lastly, the troy ounce is stated to be equal to one ounce and 50 grains avoirdupois, i.e. to 487½, instead of 480, grains."

